# Development of Registry Data to Create Interactive Doctor-Patient Platforms for Personalized Patient Care, Taking the Example of the DESTINY System

**DOI:** 10.3389/fdgth.2021.633427

**Published:** 2021-03-22

**Authors:** Arnfin Bergmann, Martin Stangel, Markus Weih, Philip van Hövell, Stefan Braune, Monika Köchling, Fabian Roßnagel

**Affiliations:** ^1^NTD Study Group, NeuroTransData GmbH, Neuburg, Germany; ^2^Clinical Neuroimmunology and Neurochemistry, Hannover Medical School, Hannover, Germany; ^3^PricewaterhouseCoopers AG, Zurich, Switzerland

**Keywords:** multiple sclerosis, real-world evidence, registry, personalized medicine, patient management

## Abstract

“Real-world evidence (RWE)” is becoming increasingly important in order to integrate the results of randomized studies into everyday clinical practice. The data collection of RWE is usually derived from large-scale national and international registries, often driven by academic centers. We have developed a digitalized doctor–patient platform called DESTINY (**D**atabas**E**-assi**S**ted **T**herapy dec**I**sio**N** support s**Y**stem) that is utilized by NeuroTransData (NTD), a network of neurologists and psychiatrists throughout Germany. This platform can be integrated into everyday practice and, as well as being used for scientific evaluations in healthcare research, can also serve as an individual, personalized treatment application. Its various modules allow for a timely identification of side-effects or interactions of treatments, can involve patients via the “My NTC Health Guide” portal, and can collect data of individual disease histories that are integrated into innovative algorithms, e.g., for the prediction of treatment response [currently available for multiple sclerosis (MS), with other indications in the pipeline]. Here, we describe the doctor–patient platform DESTINY for outpatient neurological practices and its contribution to improved treatment success as well as reduction of healthcare costs. Platforms like DESTINY may facilitate the goal of personalized healthcare.

## Introduction

A doctor's occupational image is changing. The amount of specialist medical knowledge that is now available may not even be acknowledged by experts in the field. Thus, digitalization offers ways to provide targeted support to doctors in their clinical decision-making process (1). Suitable digital systems, however, not only provide added value in everyday clinical practice, but are also an important scientific resource. The European Medicines Agency (EMA) supports the use of so-called “real-world evidence” (RWE), or “real-world data” (RWD), to overcome the division existing to date between research and practice, and to create a learning health system (2). RWE, in this view, would not replace randomized clinical trials (RCT), but instead would help to answer those questions which RCT settings alone cannot, or cannot fully answer (3).

Such support systems are particularly suited to complex chronic diseases with long clinical duration, varying manifestations, and very diverse treatment options. In the field of neurology, this is particularly true for multiple sclerosis (MS). This was recognized very early on by some stakeholders and the first digital registry databases were established at the end of the 1990s/beginning of the 2000s to document and evaluate real-world patient data from multiple practices in a standardized way (4). In recent years, large international registry databases have thus also reported important findings on disease progression and treatments for MS, which can only be learned from RWE data (5, 6). Some years ago, it was acknowledged that these registry databases could be used to develop special algorithms to help doctors take decisions about the increasingly complex range of MS treatments. This would enable them to consider cases individually and make adjustments for optimal treatment at an earlier stage (7).

Especially in outpatient settings, solutions are required which can be easily integrated into normal clinical practice, and are not unnecessarily onerous for doctors. Such systems involve (1) electronic storage of patient data and (2) the processing and display of these data, so that the doctor always has an up-to-date view of the patient's development. Based on these data an informed decision can be made on how best to treat the patient. In this way, suitable systems can provide significant added value to doctors, patients and, ultimately, the healthcare system itself, as doctors with such digital support have improved treatment outcomes and can thus reduce the costs resulting from the condition (8). Such systems make it easier to apply the concept of individualized treatment, while including and involving patients.

A prerequisite of such systems are high-quality data from everyday clinical care. In this article we provide an overview of the European MS registry data and show, using the example of the German doctors' network NeuroTransData (NTD) and the platform DESTINY (**D**atabas**E**-assi**S**ted **T**herapy dec**I**sio**N** support s**Y**stem), how such platforms can also be applied in the German healthcare context to support physicians, enhance treatment success, and reduce healthcare costs.

## European Registries and RWE Initiatives

According to the group “MS Brain Health”, an international multidisciplinary association of well-known MS experts, the key factors in successful long-term treatment of MS are: regular monitoring of disease activity, standardized recording of the data collected in suitable databases, and the use of digital systems to optimize treatment strategies and therapy decisions (9).

In many European countries there are already initiatives to meet the need for standardized recording of data. An overview of European MS registries is shown in Table 1.

**Table 1:** European MS registries [based on (10–12)].

**Country**	**Name of MS registry**	**Institution**	**Active since**	**Number of patients**
Belgium	BELTRIMS	Belgian Study Group for Multiple Sclerosis	2012	1,000
Croatia	AMSSC	Association of Multiple Sclerosis societies of Croatia	2006	2,817
Czechia	ReMuS	IMPULS	2013	10,999
Denmark	The Danish Multiple Sclerosis Registry	The Danish Multiple Sclerosis Center, Rigshospitalet, Copenhagen	1956	25,000
Finland	Finnish MS Register	Finnish Hospital Districts	2014	8,746
France	OFSEP	EDMUS Foundation, Université Claude Bernard, Hospices Civils de Lyon	1976 (2011^a^)	56,400
Germany	MS-Register der DMSG (Bundesverband e.V.)	MS Forschungs- und Projektentwicklungs-gGmbH	2001 (2014^b^)	48,000
Germany	MSDS3D	Center of Clinical Neuroscience, University Hospital Dresden	1998	>7,000
Germany	Deutschsprachiges Multiple Sklerose und Kinderwunsch Register (DMSKW)	Kerstin Hellwig	2006	1,500
Germany	REGIMS	Krankheitsbezogenes Kompetenznetz Multiple Sklerose (KKNMS)	2013	700
Germany	NTD Registry database	Ärztenetzwerk NeuroTransData	2008 (2012^c^)	25,000
Greece	The Greek MS Society	The Greek MS Society	2011	5,323
Italy	Italian MS Register	Fondazione Italiana Sclerosi Multipla (FISM)/University of Bari (UNIBA)	2001 (2016^d^)	44,894
Italy	Liguria Regional MS Registry^e^	No information provided	2012	929
Norway	Norwegian MS Registry and Biobank	Haukeland University Hospital	2001	8,500
Poland	Polish MS Registry (RejSM)	Konskie and AGH University of Science and Technology, Krakow	2011	8,845
Serbia	MS Society of Serbia	Clinic of Neurology, Faculty of Medicine, University of Belgrade and at the Clinical Centre of Serbia	1996	2,250
Spain	Epidemcat (MS Registry of Catalonia)	Department of Heath of the Government of Catalonia	2008	1,520
Sweden	Swedish Neuro Registries—MS	Karolinska University Hospital, Stockholm	1997	18,700
Switzerland	Swiss MS Cohort Study^f^	University Hospital of Basel	2012	1,200
Great Britain	UK MS Register	Swansea University	2009	16,000
Australia^g^	MSBase	MSBase Foundation Ltd.	2004	49,800

a *Start of the French OFSEP registry in its current form*.

b *Start of the new web-based data collection and of a new minimal data set*.

c *Start of web-based data collection*.

d *Start of the Italian iMedWeb database (Italian MS registry)*.

e *Until 2017, the Liguria regional MS registry was part of the Italian MS registry*.

f *One of the two Swiss MS databases; a cohort study with fixed follow-up intervals*.

g *MSBase is an association of MS expert centers in 33 countries, including in Europe, with its head office in Australia*.

With regard to the experts' call for ongoing monitoring of disease activity, the various initiatives have implemented a largely standardized system for monitoring the patient population via the MS registry databases. Many types of data are regularly entered into these registries: personal data, basic disease data, relapses, disability, cognition, treatments and therapies, magnetic resonance imaging (MRI), paraclinical measures, patient-reported outcomes, depression, fatigue, co-morbidities, socioeconomic data, societal services, and data on the use of health system resources (10).

Seven of the nineteen registries which responded to the survey carried out by Glaser et al. (10) involve patients more actively in the treatment process by the use of questionnaires, and also use these data in a clinical setting.

Systems for the automated analysis and interpretation of the data collected may help the doctor decide on the most appropriate treatment and have already been used successfully for some time in clinical practice. Table 2 shows a selection of systems developed in the context of clinical registry databases for MS patients.

**Table 2:** Digital support systems to support doctors in their therapy decisions (13–20).

**Name of the system**	**Functionality**	**Institution/Developer**	**Year**
OPTEM	Gives objective automatized recommendations for treatment optimization in MS patients based on CMSWG criteria	Canadian Multiple Sclerosis Working Group (CMSWG)	2008
EBDiMS (Evidence-Based Decision Support Tool in Multiple Sclerosis)	A prognostic calculator that delivers individualized estimates of disease progression for MS patients	Sylvia Lawry Centre for Multiple Sclerosis Research	2012
MS Curves	An online tool for assessing MS severity using the MSBase Registry	MSBase Foundation Ltd.	2012
DET (Disability Expectancy Table)	Reference table of disability outcomes in MS to compare a patient's disability to others with the same disease duration	North American Research Committee on Multiple Sclerosis (NARCOMS)	2013
Web-based decision support tool for prognosis simulation in multiple sclerosis	A web-based decision support tool to predict both the likelihood of CIS to CDMS conversion, and the long-term prognosis of disability level and SPMS conversion, as well as assess and monitor the effects of treatment	ARN—Anestesia, Reanimação e Neurologia	2014
BREMSO (Bayesian Risk Estimate for MS at Onset)	A tool which can be used in the early stages of MS to predict its evolution, supporting therapeutic decisions in an observational setting	MSBase Foundation Ltd.	2015
Function Watch	A tool to present patients data in an easy way and comparing it with the results of a matching reference group to give immediate feedback tot he clinician, if the patient performs better or worse than an average patient	Swedish Neuro Registries—MS/Karolinska University Hospital, Stockholm	2015
Automatic Classification of Multiple Sclerosis Clinical Courses	Fully automated tool that classifies Multiple Sclerosis patients into four clinical profiles using structural connectivity information	OFSEP/EDMUS Foundation, Université Claude Bernard, Hospices Civils de Lyon	2016

The tools and systems shown here use various methods to extract the data stored in the respective registry databases to display the current clinical status of individual patients. This helps the doctor to decide on treatments, and, using individual patient data, to propose further measures such as adjustment of the current therapy or further investigations.

## The Doctor–Patient Platform Destiny

The comprehensive doctor-patient platform DESTINY was developed by the NTD network with a view to provide digital support to physicians in their individual clinical decisions, using pooled clinical experience accumulated over the years.

**Figure 1:**
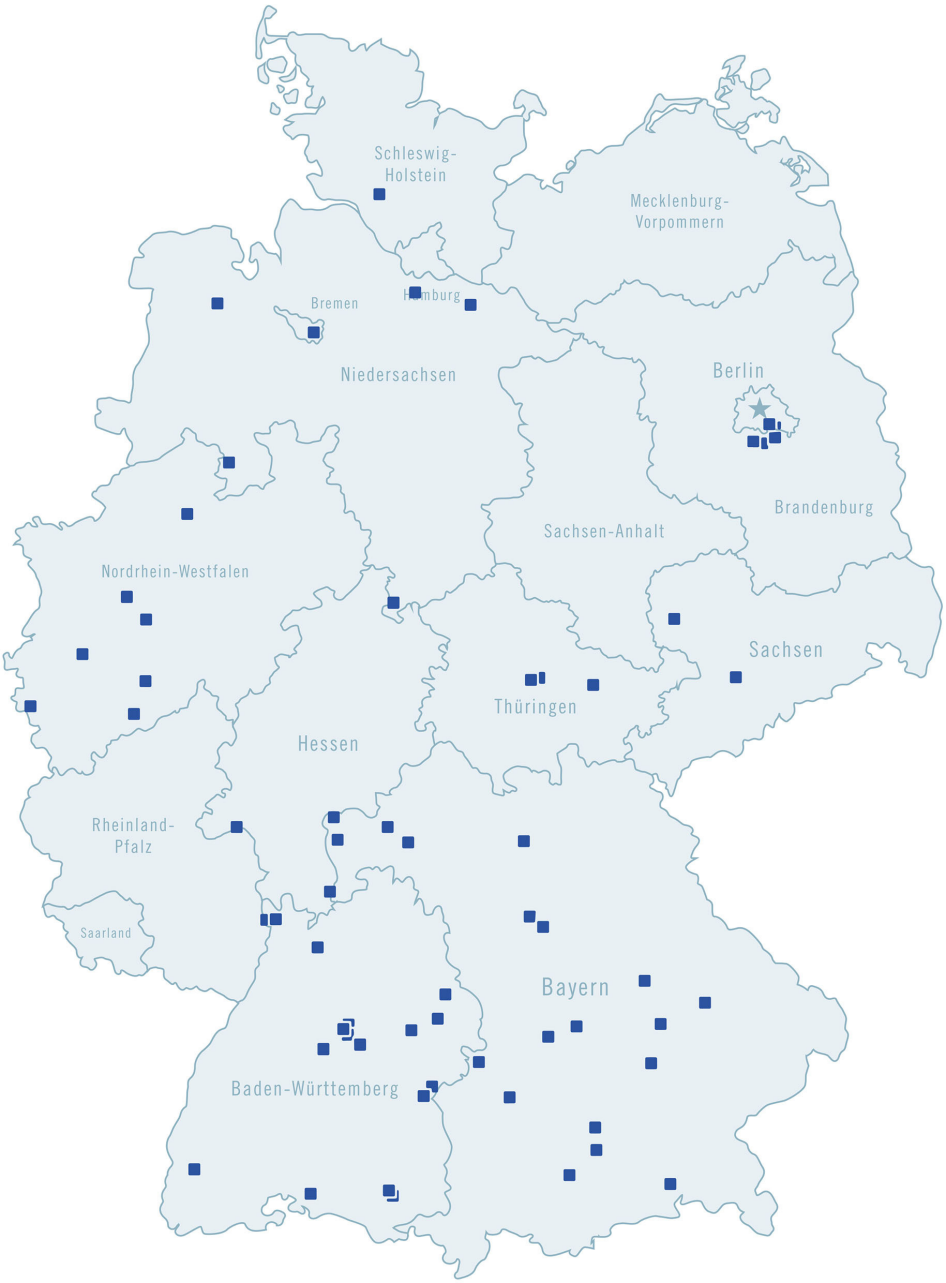
NeuroTransData member practices in Germany.

NeuroTransData (NTD) is a network of physicians in the field of neurology and psychiatry, throughout Germany. Its members are from modern and fully digitalized practices (64 centers in November 2020) located throughout the country (Figure 1). Every year, the practices in this network treat more than 600,000 patients. Since NTD was founded in 2008, pseudonymized patient data have been entered into a registry database which currently covers seven indications (movement disorders, dementia, depression, epilepsy, migraine, MS, and schizophrenia). NeuroTransData (NTD) has established a digital treatment process for its MS patients, based on the NTD registry database, which displays patient clinical progression data. At the beginning of 2018, a digital treatment process was established for migraine as a second indication.

**Figure 2:**
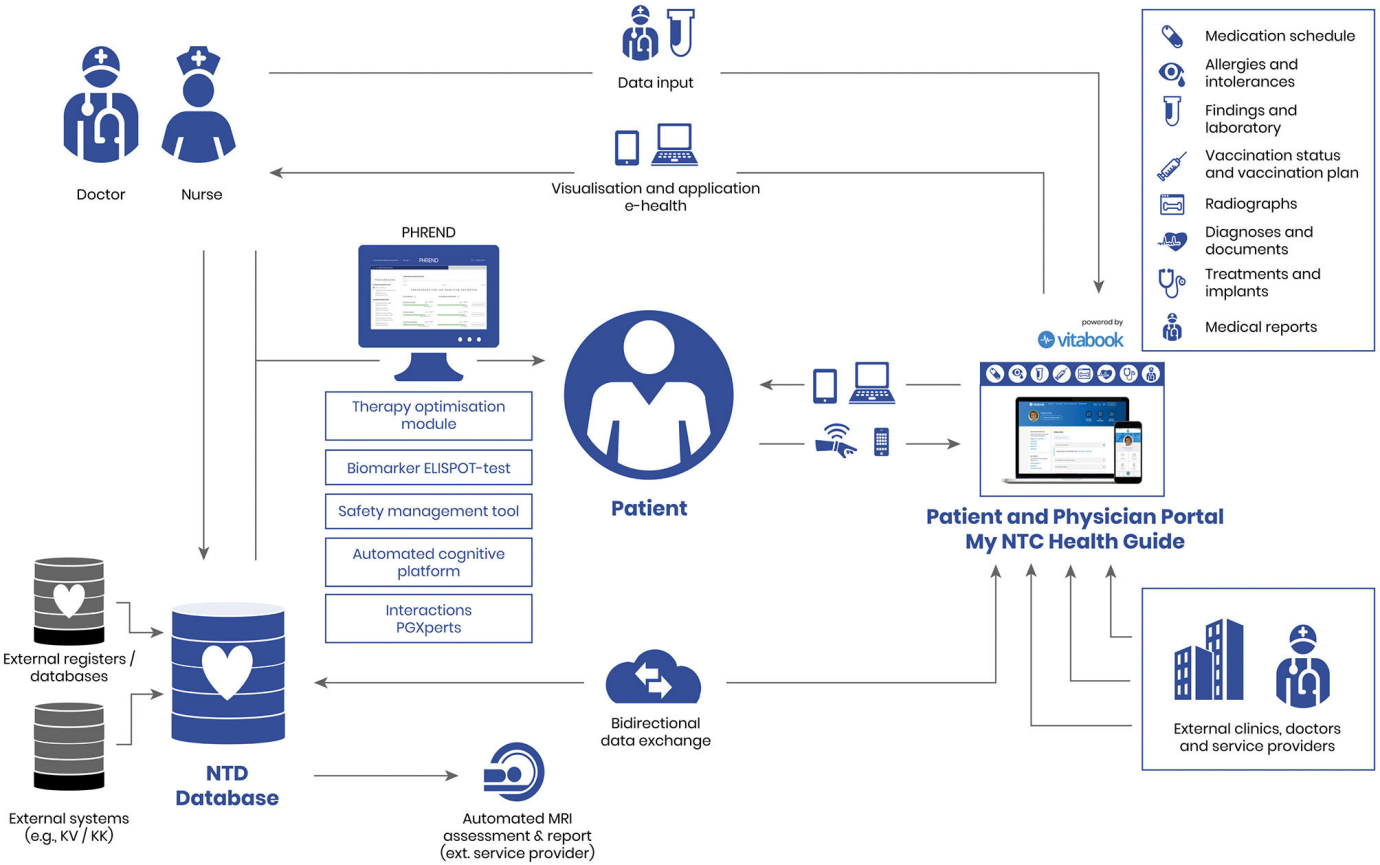
DESTINY (DatabasE-assiSted Therapy decIsioN support sYstem).

DESTINY now contains a set of personalized medicine modules for doctors and patients. At the core of DESTINY is the NTD registry database, which is linked to all modules through interfaces (Figure 2).

DESTINY has helped to achieve high-quality and transparent treatment at lower costs (fewer hospital admissions). The RWD gathered can also be used to answer important scientific questions in healthcare research (21). DESTINY, moreover, is not limited to neurology, but is also transferable to other specialist areas.

One important aspect of DESTINY is the patient portal “My NTC Health Guide” with an app on a tablet or smartphone to be used in the waiting room or at home. This portal on the one hand includes patients in therapy decisions and on the other hand allows to include patient-oriented data, such as quality of life, into the decision-making.

The following paragraphs explain the function of the various DESTINY modules. This system underpins the successful integration of digital support systems.

### Data Protection—Informed Consent and Pseudonymization Process

The NTD registry database falls under a high specification data protection system developed in conjunction with the Institute for Medical Information Processing, Biometry and Epidemiology (IBE) of the Ludwig Maximilians University of Munich (LMU). It has been inspected and approved by the Ethics Committee of the Bavarian State Chamber of Physicians and the North Rhine Chamber of Physicians. Compliance with data protection legislation, especially the Federal Data Protection Act (BDSG) and the General Data Protection Regulation (GDPR), is ensured by an appropriate informed consent and encryption process, continuously monitored and checked by an external data protection officer.

Personal data are processed on the basis of informed consent from that patient. Data are usually stored for long periods. When a patient leaves the system, no further data are collected. The data gathered up until that time are anonymized, i.e., are still used for analysis but cannot be traced back to the individual. The patient may ask for his/her data to be deleted at any time.

The following pseudonymization process is applied: The encoded patient list with identifying data (IDAT) and the healthcare database (VDB) containing medical data (MDAT) are run separately. A patient receives an identification number—the PUID (patient unique identifier)—which is stored with the IDAT in the registry database. The patient's doctor knows this number, which is used to clearly link primary data with the registry database. The patient's doctor (AID) is the only person to have access to the healthcare module with regard to the patient's treatment. The AIDs are managed in the organization database.

The IDAT are encoded and stored centrally. The symmetrical encryption procedure AES 256 is used for this, in line with recommendations from the Federal Office for Information Security. The key is only known to the patient's doctor. This means that NeuroTransData GmbH cannot decode the IDAT. The keys are administered by an external Trust Center at Ludwig Maximilians University, Munich.

### NTD Registry Database

The core of DESTINY is the NTD registry database that has been accumulating pseudonymized patient data since 2008, including diagnosis, treatment and quality of life, side-effects, and reasons for changes in treatment.

This database-assisted registry, also web-based since 2013, is a modular system containing basic documentation and a multitude of specialist modules (healthcare module, registry module, study module, patient module, administration module). All data are collected systematically in the participating practices and stored in the central database. The healthcare and registry modules have a standardized set of disease-specific basic documentation while the study module also displays additional sets of data to groups within the registry. For the purposes of treatment monitoring and support, disease progression, and treatment procedures are represented graphically for each individual patient. The administration module manages system users, practice master data, studies and metadata. The patient module allows active involvement of patients in the treatment process by the use of electronic questionnaires. Appropriate measures are taken to ensure confidentiality, integrity, availability, authenticity, reviewability, and transparency of the registry. For the purpose of data quality control, practice-oriented protocols and queries are displayed in the online registry, and are dealt with by the practices.

The registry is updated continuously, adapted and expanded to meet clinical practice requirements. Due to its modular structure, the system can easily be extended to cover other indications and areas of application.

The following indications are currently actively covered in the NTD registry database:

- Multiple sclerosis,
- Migraine,
- Epilepsy,
- Movement disorders,
- Dementia,
- Depression,
- Schizophrenia.

### Therapy Optimization Module for Multiple Sclerosis

Since 2013 the therapy optimization module has been an integral part of the NTD registry database. This module enables the continuous recording and monitoring of Expanded Disability Status Scale (EDSS) scores, relapse rate, MRI findings, quality of life, and other parameters of MS patients. If one or several of these values deteriorate, the system sends a “red flag” warning to the doctor with a recommendation that the current therapy/treatment should be reviewed. Obviously, it is still up to the patient's doctor to take the final decision.

### PHREND (Predictive Healthcare With Real-World Evidence for Neurological Disorders)

The predictive module PHREND enables the doctor to find the most suitable MS medication for an individual patient and to predict the progression of the disease if the patient is given a particular treatment (22, 23). Working with an experienced team from PricewaterhouseCoopers (PwC) AG, Switzerland, a special algorithm was developed which can use the past data in the NTD database to calculate how the disease is likely to develop in a specific patient under the influence of different treatments. The algorithm underpinning PHREND was recently published in a methodological paper (24).

**Figure 3:**
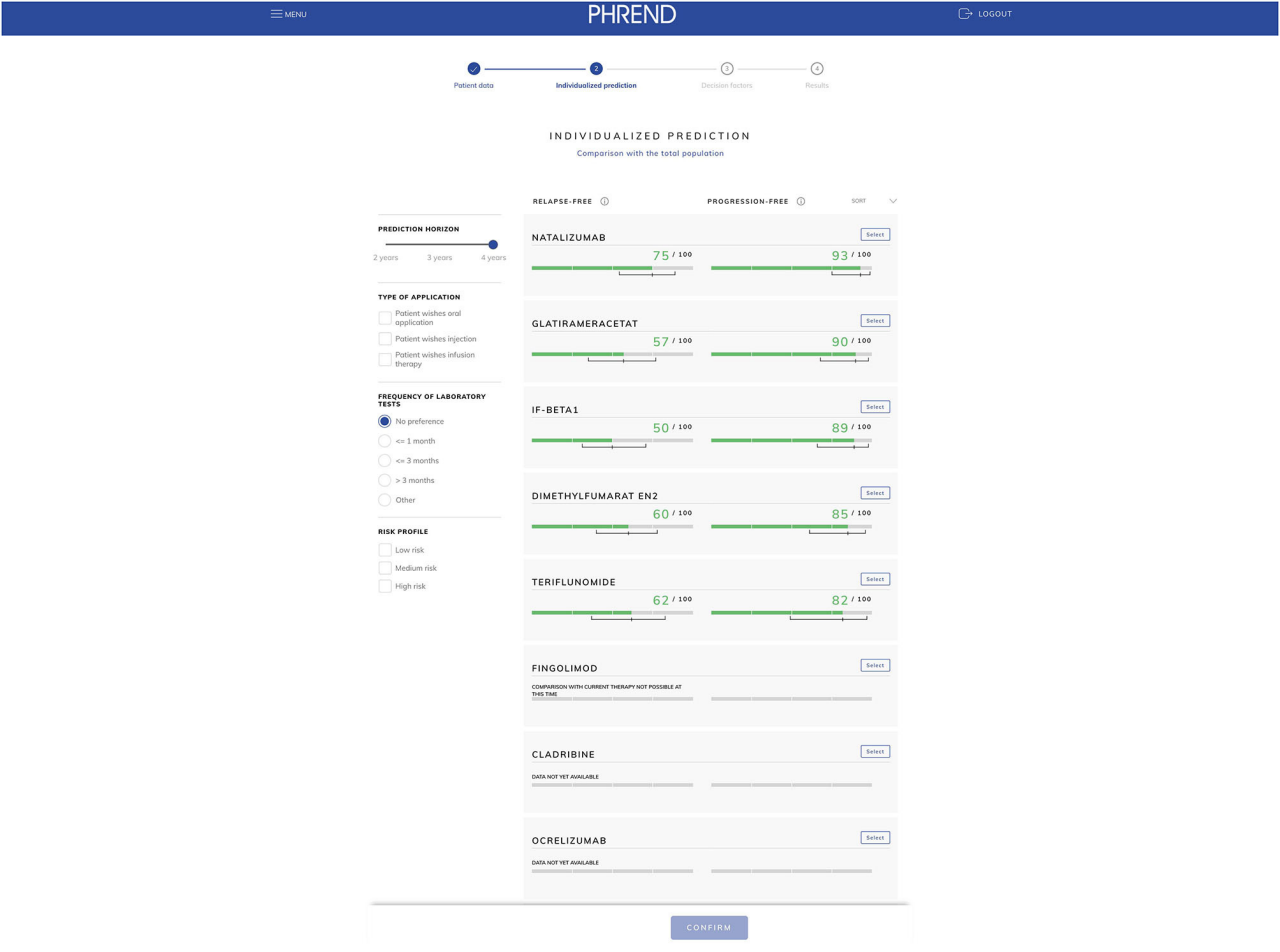
PHREND (Predictive Healthcare with Real-world Evidence for Neurological Disorders): example of an individualized treatment prognosis.

This system requires data that are known from clinical studies to determine the progression of the disease such as gender, date of birth, date of MS diagnosis, current treatment, any previous treatments, current EDSS score, date of the most recent relapse, and the number of relapses in the last 12 months. Using these data, the algorithm creates a graphical presentation showing the probability of the patient remaining relapse- and progression-free if taking various medicines over a set period of 2–4 years (Figure 3). The outcome “MRI progression-free” has been added by end of 2020. This gives the patient an independent treatment proposal with an expert prediction of how he/she may respond to the various treatments.

The therapy decision process takes account not just of the “hard facts” from the RWE data, but also of factors (preferences) important to individual patients, such as:

- pregnancy/childbearing preferences
- type of application
- possible side-effects
- frequency of laboratory tests.

After a structured discussion with the patient, the doctor and patient can together select a medicine and discuss the patient's individual wishes in more detail. The outcome of this discussion can then be emailed to the patient or printed. The patient is thus more closely and actively involved in decisions on his/her treatment.

PHREND has a CE mark as a class 1 medical device. Class 2a certification is currently underway.

### Drug-to-Drug Interaction-Check (PGXperts)

The module “PGXperts® InteraktionsCheck,” developed by HMG Systems Engineering GmbH, has been used in NTD practices since autumn 2019. This tool shows the doctor whether a planned medication interacts with any other medicines being taken by the patient. Within seconds, the module displays information on any interactions between medicines, active substances, food and habits, as well as identifying any genetic variations. The clear presentation of genetically-determined interaction risks enables rapid identification of patients who could benefit from pharmacogenetic testing.

### Patient Portal “My NTC Health Guide”

**Figure 4:**
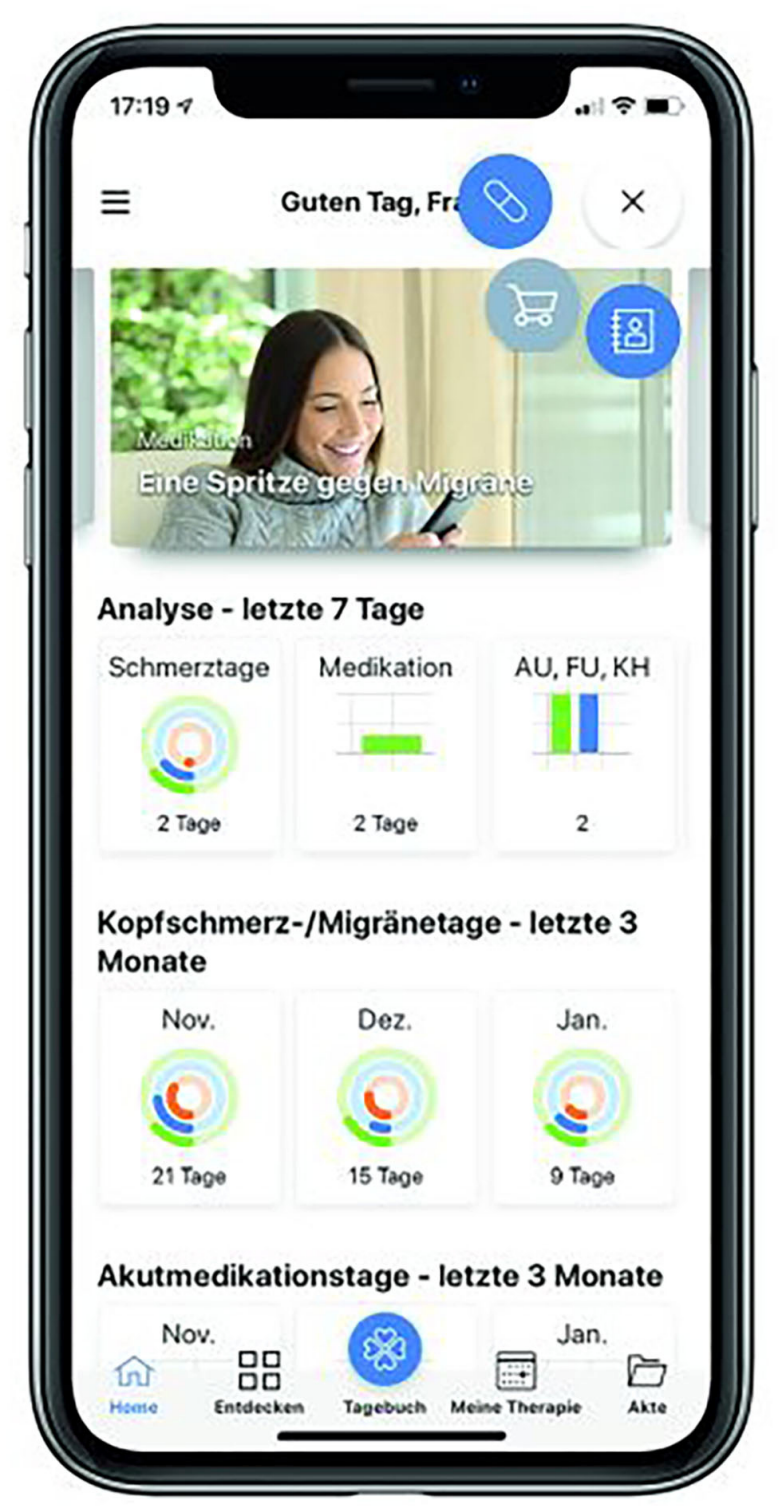
My NTC health guide for migraine (The image depicting an identifiable individual was obtained from the public database “Shutterstock”).

Another important module in DESTINY is the patient portal “My NTC Health Guide” (Figure 4). Patients can choose to use the portal on a PC, a tablet or on a smartphone app. The portal includes all content required in electronic health and patient records by Germany's Digital Healthcare Act [Digitale-Versorgung-Gesetz (DVG)] and set out in the draft Patient Data Protection Act [Patientendaten-Schutz-Gesetz (PDSG)]. It can also be used to involve patients actively in the treatment process, thus achieving better patient adherence, compliance, and empowerment. After all, insufficient adherence to treatment is one of the biggest dangers to effective treatment (25).

Studies on MS treatments show that only around 50% of MS patients take their prescribed medication in accordance with doctors' instructions (26). There are many reasons for this poor adherence, however, patient-doctor communication outside planned appointments is extremely important. This is where the “My NTC Health Guide” comes in.

Patients can use their own data to follow the course of their disease, shown graphically. They can also enter data themselves via the portal and store findings (e.g., non-medication applications such as endurance sports). For the first time, each patient has a written and graphical overview of the progress of his/her treatment. In addition, brief, non-technical articles on new developments, treatment options, and research findings are regularly posted on the patient portal by an editorial team from the NTD network, so that the patient is continuously kept informed of any new developments. This significantly strengthens the role of the patient who becomes a “contributor” rather than just an “information recipient”. One striking example of this is the headache diary developed for migraine patients: the data entered into the diary are shown graphically in the various dimensions, bidirectionally for patients and doctors (27). For example, the doctor might identify a link between regular participation in endurance sports and an easing of migraines in a patient and could optimize his/her treatment accordingly (28).

### Side-Effects Module

Adverse drug reactions (ADRs) are documented in the database by means of a special ADR form based on the reporting forms of the Federal Institute for Drugs and Medical Devices (BfArM) and the Drug Commission of the German Medical Association (DCGMA). The data are stored in the database and can also be transmitted to the relevant authorities for further follow-up.

### ACT Module (Automated Cognitive PlaTform)

The transfer of analog (paper and PDF findings) to digital data is a major problem. Until now this work has been done manually by trained workers in the NTD network, and is therefore very time-consuming and expensive. The ACT module, in addition to OCR reading techniques, uses so-called natural language processing (NLP) to target and extract the relevant information from analog medical documents (e.g., doctors' letters, laboratory findings), converting the units if necessary and transferring the data to the database in the required format.

### Future Module: Information Cockpit for Doctors

**Figure 5:**
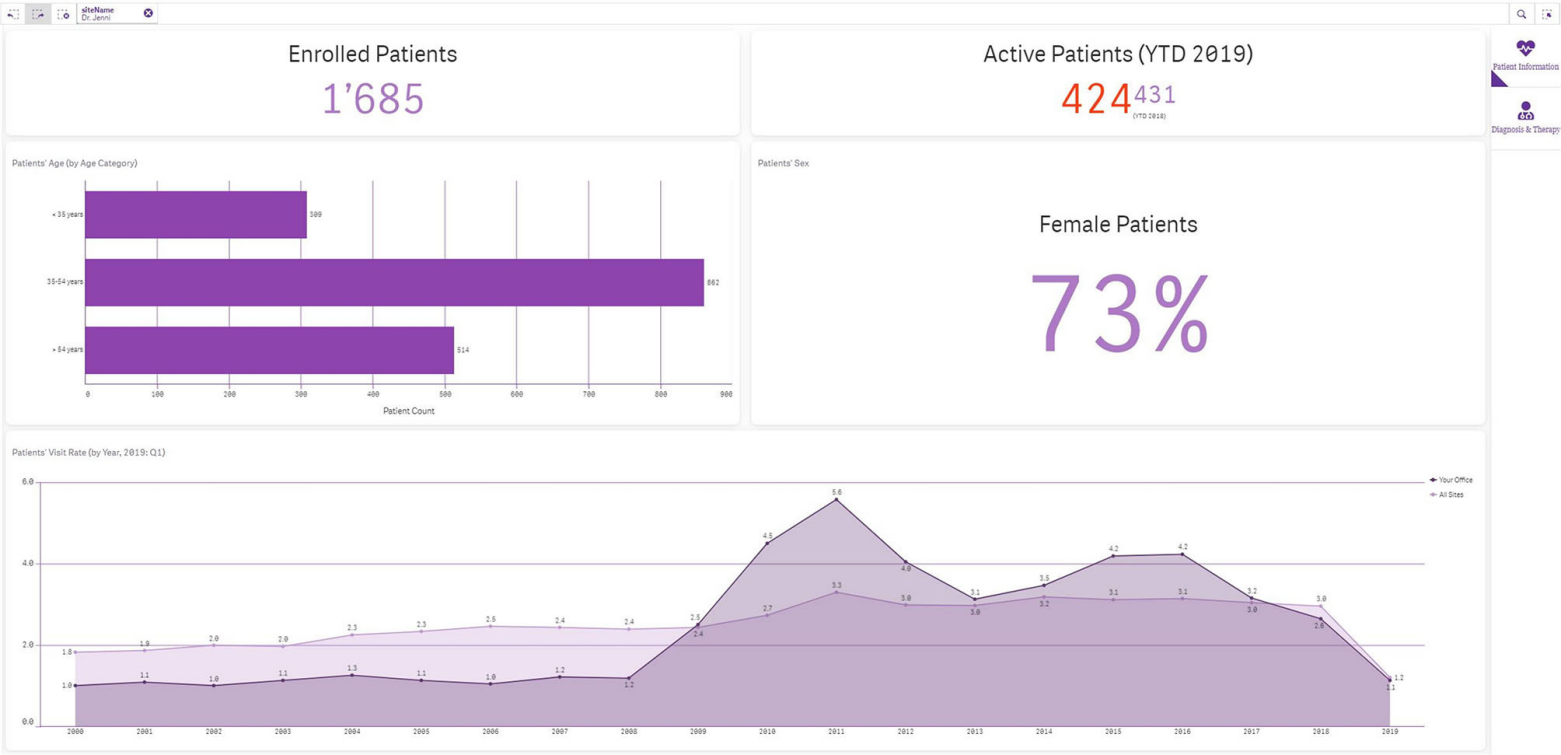
Information Cockpit for doctors.

This module will give the doctor a specific overview of the patients in his or her care, above and beyond the database indications. It can show, for example, distributions by age, gender, state of disease, and the results of clinical test procedures. The doctor can also compare his/her practice (anonymously) with other practices (benchmarking). An algorithm is also being developed which will automatically check planned prescriptions for possible “off-label use” and risk of recourse, and will generate corresponding warnings for the doctor. The information cockpit will be released in early 2021 (Figure 5).

## Usage and Effect of Destiny in the NTD Network

### Usage of Destiny in the NTD Network

**Figure 6:**
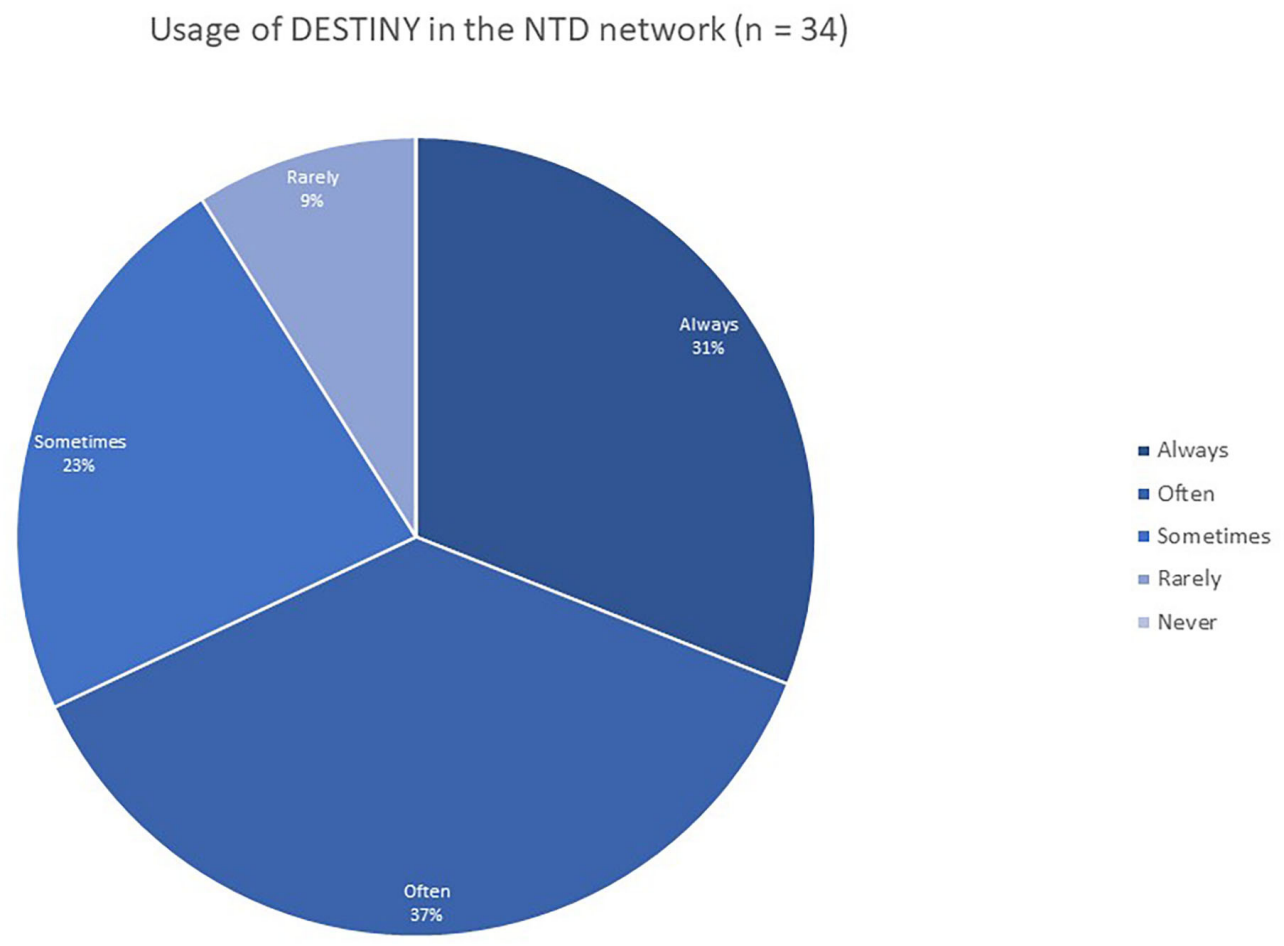
Usage of DESTINY in the NTD network.

A survey in the NTD network in 2019 (*n* = 34 practices) shows that the majority of NTD centers use DESTINY actively in their routine clinical care, with 68% of participating NTD centers using DESTINY always or often, 23% sometimes, and only 9% indicating that they rarely use the system (Figure 6).

**Figure 7:**
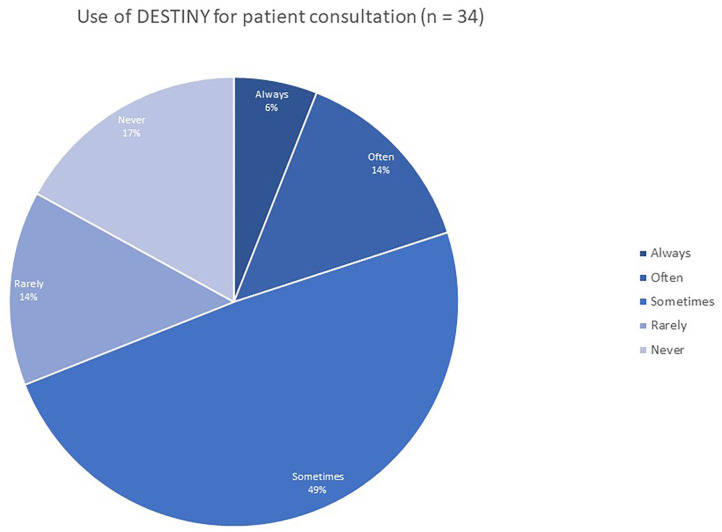
Use of DESTINY for patient consultation.

The survey also demonstrates that DESTINY is not only used by the NTD doctors for their daily routine, but also supports the doctors in their patient consultations during visits. Twenty percent of NTD doctors indicate they use DESTINY always or often during patient consultations, while 49% sometimes use the system. However, there are still 14% who only rarely use DESTINY during patient consultation and 17% never use the system while speaking to the patient (Figure 7).

### Effect of Destiny in the NTD Network

#### Methodology

To assess the effect of the digital treatment process with DESTINY on the treatment success (measured by clinical parameters) as well as on healthcare cost reduction (measured by hospitalization rate), a retrospective statistical analysis was performed.

The dataset contained 16,702 patients with remitting-relapsing multiple sclerosis (RRMS), age ≥18 years for an observation period from 01 January 2010 to 31 December 2017.

The patient population was divided into the following subgroups:

- Observation period 2010–2012: Before implementation of the digital treatment process,
- Observation period 2013–2015: Phasing-in of the digital treatment process,
- Observation period 2016–2017: After successful implementation of the digital treatment process.

Data of the subgroup from 2010 to 2012 represent the treatment situation before implementation of the digital treatment process, so that the development and effect of the introduction of DESTINY can be assessed by comparing this baseline period to the subsequent observation periods.

The analysis plan is outlined in Table 3.

**Table 3:** Statistical analysis plan.

**Parameter**	**Observation period**	**Definition**	**Evaluation**
Percentage of patients with immunomodulatory therapy	2010–2012 2013–2015 2016–2017	Immunomodulatory therapy YES for ≥3 months in index year	Number of patients, % patients
Percentage of newly diagnosed patients with first immunomodulatory therapy	2010–2012 2013–2015 2016–2017	First diagnosis of RRMS in index year AND First immunomodulatory therapy within ≤ 6 months in index year	Number of patients, % patients
Time from first symptom to initiation of immunomodulatory therapy	2010–2012 2013–2015 2016–2017	Number of days from date of first symptom diagnosis (first manifestation) to date of initiation of first immunomodulatory therapy	Descriptive evaluation (mean)
Time from therapy initiation to discontinuation	2010–2012 2013–2015 2016–2017	Time from the initiation of therapy until discontinuation in the index period in months	Descriptive evaluation (mean)
Annualized relapse rate under immunomodulatory therapy	2010–2012 2013–2015 2016–2017	Treatment with immunomodulatory therapy ≥3 months at the time of relapse	Descriptive evaluation (mean)
Time to first relapse under immunomodulatory therapy	2010–2012 2013–2015 2016–2017	Treatment with immunomodulatory therapy ≥3 months at the time of relapse Time from initiation of therapy until first relapse in index period in days	Descriptive evaluation (mean)
Time from first symptom to confirmed disability progression according to EDSS-score	2010–2012 2013–2015 2016–2017	Confirmed disability progression: Increase of EDSS score ≥1, 0 points compared to previous EDSS score, confirmed by EDSS measurement with same or worse score within 4–8 months At the time of first assessment of disability progression ≥3 months under treatment with immunomodulatory therapy	Descriptive evaluation (mean)

#### Results for Clinical Parameters (Table 4)

After successful implementation of the digital treatment process with DESTINY a higher percentage of patients was treated with immunomodulatory therapies (increase from 68.4 to 76.8%) with the majority of newly diagnosed patients having a treatment initiation within the first 6 months (increase from 61.5 to 69.2%). Furthermore, immunomodulatory therapy was initiated much earlier after detection of first symptoms (reduction from 233.6 to 114.2 days). Time from therapy initiation to therapy discontinuation was reduced from 22.8 months to only 6 months. Annualized relapse rate decreased from initially 0.25 to 0.16 relapses per year, while the time to first relapse increased from 322 to 720.2 days. Confirmed EDSS progression also clearly improved during the observation period: Time to confirmed EDSS progression with a baseline EDSS <3 increased from 130.7 to 169.8 months. For the group with a baseline EDSS <5 an increase from 193.4 to 243.4 months can be observed.

**Table 4:** Results for clinical parameters.

	**No. of patients**	**2010–2012**	**2013–2015**	**2016–2017**
Patients with immunomodulatory therapy (%)	12,534	68.4	75.1	76.8
Newly diagnosed patients with first therapy after ≤ 6 months (%)	4,188	61.5	71.7	69.2
Time from first symptom to initiation of immunomodulatory therapy (days)	1,880	233.6	149.4	114.2
Time from therapy initiation to discontinuation (months)	7,291	22.8	13.6	6.0
Annualized relapse rate (ARR)	14,846	0.23	0.17	0.16
Time to first relapse (days)	4,143	322.0	495.6	720.2
Time to confirmed EDSS progression with baseline EDSS <3 (months)	161	130.7	131.6	169.8
Time to confirmed EDSS progression with baseline EDSS <5 (months)	53	193.4	182.3	243.4

#### Results for Hospitalization Rate and Healthcare Costs

The hospitalization rate within the analyzed MS population was considerably reduced from 8.69% to only 3.72% during the observation period (Table 5).

**Table 5:** Results for hospitalization rate.

	**No. of patients**	**2010–2012**	**2013–2015**	**2016–2017**
Hospitalization rate (%)	11,307	8.69	6.20	3.72

Hospitalization rate in MS patients in Germany has been assessed in only a few studies with results ranging from 14.6% and 14.7% in publications from 2014 (29, 30) to 9.7% in a 2017 publication (31). This indicates that the hospitalization rate has generally improved over the years. This gradual improvement can also be observed for the MS patient population in the NTD network, whereas hospitalization rate in the NTD network is substantially lower than in the studies cited above.

Depending on the source of information, mean annual inpatient treatment costs vary from 3,203 € to 4,232 € and constitute the second largest pool of costs within the direct medical costs for MS patients (32). Flachenecker et al. indicate the healthcare costs for inpatient care with a range from 1,236 € for mild cases and 3,390 € for moderate cases to 6,783 € for severe cases (31).

Taking the healthcare costs from the literature, the cost savings through reduction of hospital admissions in the NTD network can be calculated as follows for the period of 2016–2017 (Table 6).

**Table 6:** Calculation of healthcare cost reduction.

	**NTD with average hospitalization rate**	**NTD with actual hospitalization rate**	**Cost reduction**
No. of patients	11,307	11,307	
Hospitalization rate	9.7%	3.72%	
Annual inpatient costs per patient (min.)	3,203 €	3,203 €	
Annual inpatient costs per patient (max.)	4,232 €	4,232 €	
Hospital admissions 2016 to 2017 (mean)	1,097	421	
Annual inpatient costs total (min.)	3,513,691 €	1,348,463 €	2,165,228 €
Annual inpatient costs total (max.)	4,642,504 €	1,781,672 €	2,860,832 €

Without the digital treatment process, the annual inpatient costs would have ranged from 3,513,691 € to 4,642,504 €, assuming an average hospitalization rate of 9.7%. Taking the actual hospitalization rate of 3.72%, the costs for the period 2016–2017 effectively ranged from 1,348,463 € to 1,781,672 €. The annual cost reduction in the NTD network therefore ranges from 2,165,228 € to 2,860,832 € for the period 2016–2017.

## Discussion

In recent years it has become clear that evaluation of RWE can close an important gap between randomized clinical studies and everyday application. These data have also become increasingly significant for authorities such as the EMA. The data collected come from the registries established in many countries over the last decades. Until now, however, these data have essentially only been used for scientific evaluations. We are convinced that intelligent linking of the registry data with individual patient data can also provide great added value to treatments and therapy decisions. This, however, requires a system which can be integrated into everyday practice. The digital doctor-patient platform DESTINY was set up in recent years for this very purpose—to help neurologists and psychiatrists in their daily work. It was developed as a “doctor's” tool for both doctors and patients. Initially, it was only used to document the outcomes of treatments, but over the years the experience gained was used to develop further useful instruments to optimize diagnosis, therapies, and treatments. Patients and doctors benefit directly from the joint documentation and processing of data in the various DESTINY modules and programs, in the form of individualized and improved quality of care. This concept—multilateral linking of data—is the basic principle underpinning sustained, expert collection and use of data. When registry databases are flexibly structured and operated, they can show almost immediately any adjustments which need to be made to treatments and decisions which are becoming increasingly complex. DESTINY is thus clearly far more than just a collection tool for data. By its innovative algorithms and intelligent links that use past experience it may facilitate personalized medicine. It enhances patient safety by quick identification of potential side-effects or interactions, and optimizes treatment by predictive modeling. The evaluations and prognoses made are not purely based on randomized studies but also on past clinical practice experience with all the difficulties linked to comorbidities and co-medication rarely included in randomized studies.

The survey conducted in the NTD network demonstrates the high acceptance of DESTINY and its use in routine clinical care for the benefit of both doctor and patient. The results of the retrospective analysis show a significant improvement in clinical parameters for MS patients through implementation of the digital treatment process. Furthermore, the evaluation shows a significant contribution to cost savings by reduction of hospital admissions. These results provide evidence that DESTINY successfully supports doctors to better monitor the clinical course of their patients, find the therapy best suited to the individual characteristics of the patient and eventually improve treatment success and reduce healthcare costs considerably.

This research work can serve as a starting point for further analyses and studies to investigate the effect of digital support systems on treatment success.

As stated at the beginning, a doctor's occupational image is changing. Digital systems are more and more opening up new possibilities for personalized medicine and individual treatment of patients and the role of the doctor will change accordingly. The treating physician will be provided with a broad array of support options for individual treatment decisions, and this in turn means higher treatment success for the patient.

The dataset of this analysis consists exclusively of data assessed during routine clinical care in centers of the NTD network. Although quality of data is ensured through state-of-the-art measures, over- or underestimations due to errors or bias caused by data entry and documentation by doctors or medical personnel cannot be excluded.

Due to the descriptive nature of the analysis, only assertions regarding distribution and condition of the analyzed subgroups within the sample can be made. No claim for representativeness for MS populations or outpatient neurological practices outside of this analysis can be made. Transfer to other use cases or the deduction of general statements are excluded as well.

Additionally, there are some content-related limitations which have to be taken into account: The observation period of the first two subgroups (2010–2012 and 2013–2015) differs from the observation period of the last subgroup (2016–2017). This distribution was chosen for reason of better comparison with the values mentioned in the reference publications. Over- or underestimations caused by the non-uniformity of the subgroups can therefore not be excluded.

Factors influencing the treatment success beside the digital treatment process with DESTINY (e.g., more therapy options over the years, improvement in healthcare standards, high number of MS specialist centers in the NTD network) were not taken into account by the analysis. Further research is needed to more specifically determine the effects of the digital treatment process with DESTINY on treatment success. An adequate setting could be that of a prospective study comparing the effects on treatment success and healthcare costs during a longer observation period, e.g., using two groups of outpatient sites (with and without DESTINY).

The strength of this analysis is first of all the data set which formed the basis for the analysis. The data consist of RWD that were assessed and documented in outpatient neurological practices during routine clinical care. The clear distinction of the period before the implementation of DESTINY from the final phase with full integration of DESTINY allows for a detailed analysis of the contribution the digital treatment process had for both the treatment success and the reduction of healthcare costs. Influence on the results due to deviations, errors or outliers is reduced by the sample size and the geographical distribution of sites throughout Germany. Moreover, the participating sites remained mostly the same over the complete observation period, so that bias due to a change in treatment patterns or patient population characteristics is reduced as well.

This review delivers first insights into the effect of the integration of digital systems and their use in routine clinical care in outpatient medical practices—an area where significantly more research is needed, especially when considering its social and political relevance.

In conclusion, NTD is constantly working to improve DESTINY, to refine the existing components and to integrate new modules, and making it practical and user-friendly. Currently, the platform is essentially used only within the NTD network. However, DESTINY is to be further extended in various stages to cover other indications and specialist areas and will be made available to doctors outside the network. In summary, the digital doctor–patient platform DESTINY is an instrument which has been further developed from its original function as a pure database for scientific evaluations into a tool for personalized medicine. We think that the data collected and processed can help to improve the care of individual patients and allow for a best possible usage of the healthcare system's limited financial resources.

## Author Contributions

All authors listed have made a substantial, direct and intellectual contribution to the work, and approved it for publication.

## Conflict of Interest

AB has received consulting fees from advisory board, speaker, and other activities for NeuroTransData; project management and clinical studies for and travel expenses from Novartis and Servier. MS has received honoraria for consulting, clinical studies, and lectures from Alexion, Biogen, Celgene, CSL Behring, Grifols, IGES Institut, Janssen, Merck Serono, NeuroTransData, Novartis, Roche, Sanofi-Genzyme, Takeda, Bayer Vital GmbH, MediDidac, and Teva GmbH. MW has received consulting fees from advisory board, speaker, and other activities for NeuroTransData and Novartis. PVH is employee of PricewaterhouseCoopers and contracted to perform statistical analyses for NeuroTransData. SB has received honoraria from Kassenärztliche Vereinigung Bayern and HMOs for patient care; honoraria for consulting, project management, clinical studies, and lectures and from Biogen, Lilly, MedDay, Merck, NeuroTransData, Novartis, Roche, and Thieme Verlag; honoraria and expense compensation as board member of NeuroTransData. MK has received consulting fees from advisory board, speaker, and other activities for Janssen-Cilag, NeuroTransData, and Novartis. FR is employed by the company NeuroTransData GmbH. The authors declare that this study received funding from NeuroTransData GmbH. The funder had the following involvement with the study: decision to publish, preparation of the manuscript.
